# Risk factors for juvenile idiopathic arthritis: exposure to tobacco and environmental factors during and before pregnancy

**DOI:** 10.1186/1546-0096-12-S1-P27

**Published:** 2014-09-17

**Authors:** Camila MP França, Adriana M Sallum, Clovis AA Silva, Nadia E Aikawa, Alfésio L Braga, Sylvia C Farhat

**Affiliations:** 1 Pediatric Rheumatology Unit, Faculdade de Medicina da Universidade de São Paulo, São Paulo – SP, Brazil; 2 Environmental Epidemiology Study Group, Laboratory of Experimental Air Pollution, Faculdade de Medicina da Universidade de São Paulo, São Paulo – SP, Brazil

## Introduction

Juvenile idiopathic arthritis (JIA) is a heterogeneous group of chronic arthritis that occurs in susceptible subjects and may be related to environmental triggers. In this regard, air pollution could be a potential contributor to autoimmune diseases onset and flare, as it has been recently reported by our group in juvenile systemic lupus erythematosus and dermatomyositis patients, and was rarely studied in JIA population.

## Objectives

To evaluate the influence of exposures to inhaled environmental factors at the same time during pregnancy and one year before pregnancy on JIA diagnosis in residents of a large city.

## Methods

A case-control study comprising 21 JIA and 40 controls matched by age and gender, residents in the metropolitan region of São Paulo. A structured and reliable questionnaire (kappa index for test-retest of 0.81) assessed demographic data and environmental inhalation exposure during pregnancy (occupational exposure to demolition, chalk, construction and/or quarry dust, paints, varnish, gasoline vapor and/or battery fluids; the presence of industrial activities or gas station near the mother's home/work, maternal tobacco exposure and exposure to tropospheric pollutants). Daily concentrations of inhaled particulate matter (PM10), sulphur dioxide (SO2), nitrogen dioxide (NO2), ozone (O3), and carbon monoxide (CO) were evaluated throughout one year in the pre-gestational and gestational period.

## Results

The mean current age was similar in JIA and controls (11.52±3.78 vs.11.63±3.20 years, p=0.93). “Fetal smoking” (mother’s second and smoke exposure and/or smoking mothers at home) one year before pregnancy was significantly higher in JIA patients *versus* controls (52% vs. 20%, p=0.01). Otherwise, the presence of industrial activities or gas station at mother’s work place > 200 meters was significantly lower in JIA group (14% vs. 52%, p=0.003). In univariate logistic regression models for one year before pregnancy, “fetal smoking” was significantly associated with JIA (OR4.4 CI95%1.4-14.0, p=0.012, respectively), while the presence of industrial activities or gas station at mother’s work > 200 meters had a significantly negative association with JIA (OR0.14 CI95% 0.4-0.6, p=0.006). Regarding tropospheric pollutants no positive association was evidenced (p>0.05). In multivariate analysis for one year before pregnancy, “fetal smoking” remained risk factor for JIA (OR3.69 CI95%1.06-12.8, p=0.04) and the presence of industrial activities or gas station at mother’s work > 200 meters was protective factor (OR0.16 CI95%0.4-0.7, p=0.01). Regarding pregnancy, “fetal smoking” was significantly higher in JIA group (52% vs. 20%, p=0.01), whereas maternal occupational exposure, the presence of industrial activities or gas station at mother’s work place > 200 meters and the presence of industrial activities or gas station at mother’s home > 200 meters were significantly higher in controls (9% vs. 12%, p=0.016; 19% vs. 50%, p=0.02; 52% vs. 85%, p=0.008; respectively). In multivariate analysis during pregnancy, only the presence of industrial activities or gas station at > 200 meters had a significantly association with JIA (OR0.13 CI95%0.3-0.7, p=0.02).

## Conclusion

Mother's exposure to tobacco smoking may contribute to JIA onset, while exposure to long distance industrial activities or gas station could be a protective factor for this inflammatory chronic disease.

## Disclosure of interest

None declared.

